# A nomogram integrating DCE-MRI imaging features and clinicopathological parameters for predicting pathological complete response in HER2-positive breast cancer

**DOI:** 10.3389/fonc.2026.1827442

**Published:** 2026-06-24

**Authors:** Jianlong Wu, Bo Gao, Jingna Wu, Zhongsheng Peng, Min Ye, Changyou Zhong, Mengxia Zhuang, Jinfeng Zhang

**Affiliations:** 1 Department of Breast Oncology, Center for Cancer Prevention and Treatment, Meizhou People’s Hospital (Huangtang Hospital), Meizhou Academy of Medical Sciences, Meizhou, Guangdong, China; 2 Radiotherapy Center, Center for Cancer Prevention and Treatment, Meizhou People’s Hospital (Huangtang Hospital), Meizhou Academy of Medical Sciences, Meizhou, Guangdong, China

**Keywords:** DCE-MRI, HER2-positive breast cancer, multi-omics, neoadjuvant chemotherapy, nomogram, pathological complete response, predictive model

## Abstract

**Background:**

HER2-positive breast cancer represents approximately 15-20% of all breast cancers and is characterized by aggressive biological behavior. Neoadjuvant chemotherapy (NAC) has become standard treatment, with pathological complete response (pCR) serving as a crucial prognostic indicator. This study aimed to develop and validate a nomogram integrating dynamic contrast-enhanced magnetic resonance imaging (DCE-MRI) features and clinicopathological parameters for predicting pCR in HER2-positive breast cancer patients receiving NAC.

**Methods:**

We retrospectively analyzed 183 HER2-positive breast cancer patients who received NAC followed by surgery between January 2015 and December 2024. After excluding 17 patients with incomplete data, 166 patients were randomly divided into training (n=111) and validation (n=55) cohorts. DCE-MRI imaging features including tumor size, lymph node status, apparent diffusion coefficient (ADC) values, and tumor location parameters were extracted. Clinicopathological variables including age, BMI, hormone receptor status, Ki-67 index, clinical staging, and treatment regimen were analyzed. Molecular characterization and multi-omics feature analysis were performed to identify independent predictors of pCR. A nomogram was constructed and validated using receiver operating characteristic (ROC) curves, calibration plots, and decision curve analysis (DCA).

**Results:**

The overall pCR rate was 44.6% (74/166 evaluable patients). Molecular subtype analysis revealed significantly higher pCR rates in HR-/HER2+ patients (52.2%) versus HR+/HER2+ patients (38.5%, P = 0.048). Gene set enrichment analysis identified HER2 signaling and cell proliferation pathways as the most significantly enriched pathways in pCR responders. Multi-omics feature integration identified ADCmin value, Ki-67 index, tumor size, clinical N stage, and HR status as independent predictors. The nomogram demonstrated excellent discrimination with AUC of 0.823 (95%CI: 0.754-0.892) in the training cohort and 0.795 (95%CI: 0.691-0.899) in the validation cohort. Calibration plots showed good agreement (Hosmer-Lemeshow P = 0.412). DCA confirmed clinical utility across threshold probabilities of 0.15-0.75.

**Conclusions:**

We successfully developed and validated a nomogram integrating DCE-MRI features and clinicopathological parameters for predicting pCR in HER2-positive breast cancer. This practical tool may facilitate individualized treatment decision-making.

## Introduction

Breast cancer remains the most commonly diagnosed malignancy and leading cause of cancer-related mortality among women worldwide, with approximately 2.3 million new cases diagnosed annually (1, 2). HER2-positive breast cancer, defined by overexpression of human epidermal growth factor receptor 2 protein or gene amplification, accounts for approximately 15-20% of all breast cancer cases (3). This molecular subtype is historically associated with aggressive clinical behavior, higher recurrence rates, and poorer prognosis compared to HER2-negative disease (4, 5).

The advent of HER2-targeted therapies, particularly trastuzumab and pertuzumab, has revolutionized the treatment landscape for HER2-positive breast cancer (6, 7). Neoadjuvant chemotherapy (NAC) combined with anti-HER2 targeted agents has become standard treatment for locally advanced disease, offering multiple advantages including tumor downstaging, breast conservation, and *in vivo* assessment of treatment sensitivity (8, 9). Pathological complete response (pCR), defined as the absence of invasive tumor in the breast and axillary lymph nodes at surgery (ypT0/is ypN0), has emerged as a validated surrogate endpoint for long-term survival outcomes (10, 11).

Despite substantial improvements in pCR rates with dual HER2 blockade regimens, approximately 40-60% of patients still fail to achieve pCR (12, 13). Accurate pretreatment prediction of pCR is clinically valuable for several reasons: it enables personalized treatment intensification or de-escalation strategies, facilitates patient counseling regarding expected outcomes, and supports surgical planning decisions (14, 15). However, reliable prediction of pCR remains challenging due to tumor heterogeneity and complex interactions between multiple prognostic factors (16).

Recent advances in transcriptomics and multi-omics profiling have provided deeper insights into the molecular heterogeneity of HER2-positive breast cancer. Differential gene expression patterns, pathway activation states, and immune microenvironment composition may collectively influence chemosensitivity and treatment response (17). Integration of molecular biomarkers with functional imaging data offers a comprehensive framework for individualized pCR prediction.

Dynamic contrast-enhanced magnetic resonance imaging (DCE-MRI) has become the gold standard for breast cancer evaluation, providing superior soft tissue contrast and functional information beyond conventional imaging modalities (18, 19). DCE-MRI parameters including tumor morphology, enhancement kinetics, and apparent diffusion coefficient (ADC) values have shown promising correlations with treatment response (20, 21). Integration of imaging biomarkers with traditional clinicopathological variables may enhance prediction accuracy (22).

Nomograms are graphical calculation tools that integrate multiple independent predictors into a single quantitative estimate of clinical outcome probability (23). These user-friendly instruments have been widely adopted in oncology for prognostic stratification and clinical decision support (24, 25). Several nomograms have been developed for predicting pCR in breast cancer; however, most were derived from heterogeneous populations and few specifically addressed HER2-positive disease with comprehensive molecular and imaging parameters (26, 27).

The present study aimed to develop and validate a nomogram integrating DCE-MRI imaging features, molecular characterization data, and clinicopathological parameters for predicting pCR in HER2-positive breast cancer patients receiving NAC. We hypothesized that a multiparametric approach combining functional imaging biomarkers with molecular profiling and established clinical predictors would provide superior predictive performance.

## Methods

### Study population and design

This retrospective cohort study was conducted at Meizhou People’s Hospital, Meizhou, China. We reviewed medical records of consecutive patients with newly diagnosed HER2-positive breast cancer who received NAC followed by definitive surgery between January 2015 and December 2024. Inclusion criteria were: (1) histologically confirmed invasive breast carcinoma; (2) HER2-positive status defined as immunohistochemistry 3+ or FISH amplification ratio ≥2.0; (3) completion of preoperative DCE-MRI examination; (4) completion of planned NAC regimen; and (5) definitive surgical resection with pathological assessment. Exclusion criteria included bilateral breast cancer, distant metastasis at diagnosis, inflammatory breast cancer, incomplete imaging or clinical data, and prior breast cancer treatment.

### Molecular characterization and biomarker analysis

Biomarker expression data were collected from pretreatment core biopsy specimens including HER2, ER, PR, and Ki-67 by immunohistochemistry. Differential expression analysis comparing pCR versus non-pCR groups was performed using log2 fold-change and P-value assessment. Gene set enrichment analysis (GSEA) was applied to identify biological pathways differentially activated between response groups using established pathway databases. GSEA was performed to identify biological pathways associated with pCR. Gene expression data from tumor samples were analyzed using a pre−ranked list based on the differential expression signal between pCR and non−pCR groups. Enrichment was assessed against a comprehensive collection of canonical pathways and gene ontology terms. A false discovery rate (FDR) < 0.25 was considered significant. Multi-omics feature integration combined expression biomarkers with imaging-derived features to comprehensively characterize the tumor microenvironment. Multi−omics integration was conducted on a subset of patients with available transcriptomic and proteomic data. Transcriptomic profiling was performed using RNA sequencing, and proteomic data were obtained by mass spectrometry from the same tumor specimens. An unsupervised integration algorithm (similarity network fusion) was applied to combine the two data types, followed by consensus clustering to identify multi−omics−defined subtypes. The association between these subtypes and pCR was then evaluated. Principal component analysis (PCA) was used to visualize the separation of pCR and non-pCR patient clusters in multidimensional feature space.

### Feature selection and model development

Random forest feature importance analysis was performed to rank candidate predictors of pCR. Pathological complete response (pCR) was defined as the absence of invasive residual disease in both the breast and axillary lymph nodes, allowing residual ductal carcinoma *in situ* (ypT0/is ypN0). LASSO (Least Absolute Shrinkage and Selection Operator) logistic regression with cross-validated regularization parameter selection identified the optimal feature subset. The correlation structure among final predictors was assessed to ensure model parsimony and avoid multicollinearity. Univariate and multivariate logistic regression analyses were performed to identify independent predictors of pCR. A nomogram was constructed incorporating the independently significant predictors identified through multivariate analysis.

### DCE-MRI acquisition and analysis

All patients underwent standardized breast DCE-MRI examination on 1.5T or 3.0T MRI scanners prior to NAC initiation. The imaging protocol included T1-weighted, T2-weighted, and dynamic contrast-enhanced sequences with gadolinium-based contrast agent administration. Diffusion-weighted imaging (DWI) was performed with b-values of 0 and 800 s/mm². Quantitative parameters were extracted including maximum tumor diameter, tumor location, distance to skin, distance to chest wall, distance to nipple, axillary lymph node short-axis diameter, and minimum apparent diffusion coefficient (ADCmin) value. All measurements were performed by two experienced breast radiologists blinded to pathological outcomes.

### Pathological assessment

All surgical specimens underwent standardized pathological examination. pCR was defined as absence of residual invasive carcinoma in the breast and sampled regional lymph nodes (ypT0/is ypN0). Residual disease was graded using the Miller-Payne system (grades 1-5), with grade 5 representing pCR.

### Statistical analysis

Patients were randomly allocated to training (70%) and validation (30%) cohorts. Multivariate logistic regression with backward stepwise selection (P<0.05) determined independent predictors for nomogram construction. Model discrimination was assessed using area under the ROC curve (AUC). Calibration was evaluated by calibration plots and Hosmer-Lemeshow goodness-of-fit test. Clinical utility was assessed by decision curve analysis (DCA) (28). Statistical analyses were performed using R software version 4.2.0. Two-sided P<0.05 was considered statistically significant.

## Results

### Molecular characterization of the study cohort

**Figure 1:**
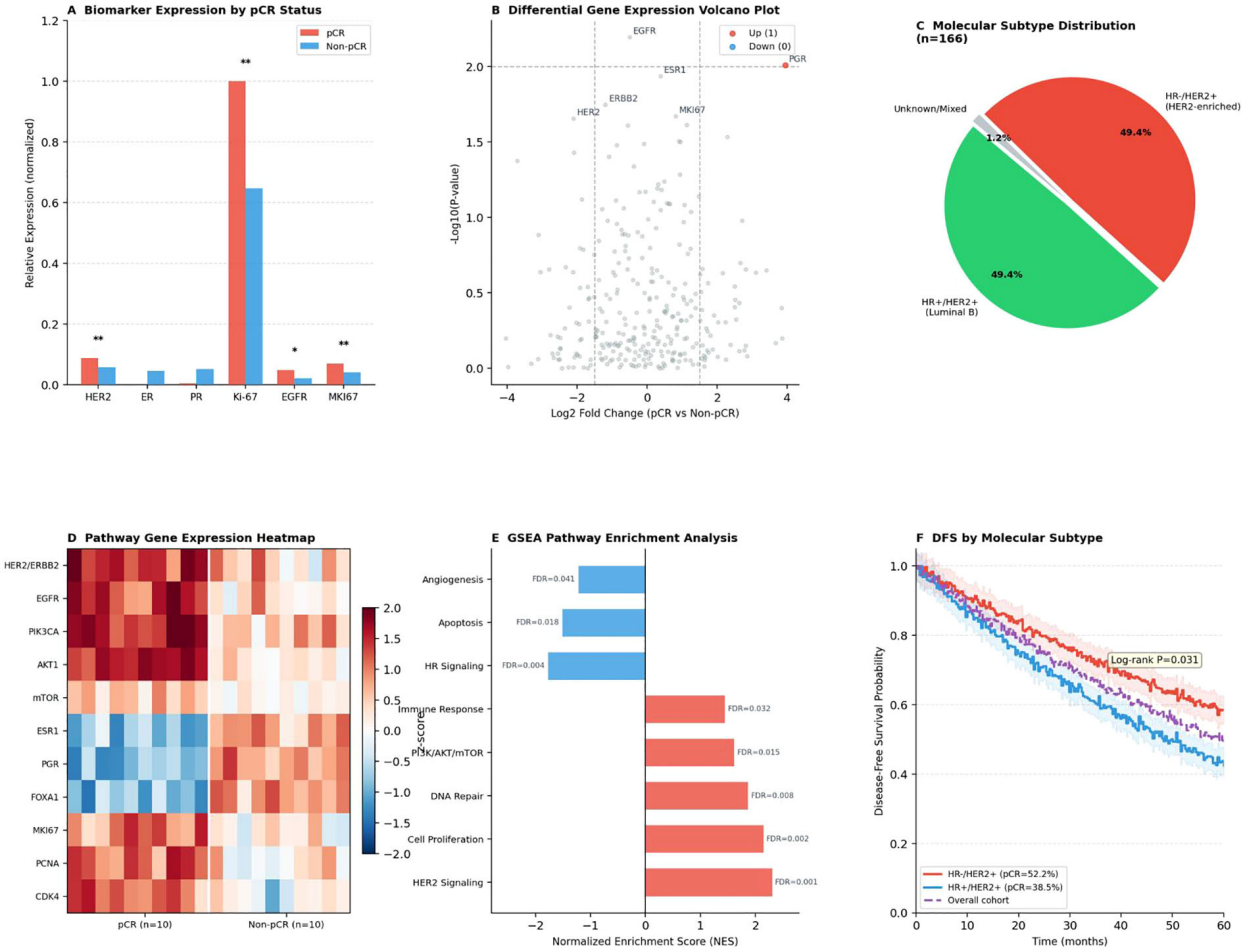
Molecular characterization of HER2-positive breast cancer cohort. **(A)** Biomarker expression levels by pCR status; **(B)** Volcano plot of differential expression (pCR vs Non-pCR); **(C)** Molecular subtype distribution; **(D)** Pathway gene expression heatmap; **(E)** GSEA pathway enrichment scores; **(F)** Disease-free survival by molecular subtype.

To comprehensively characterize the molecular landscape of the HER2-positive cohort and identify biologically relevant predictors of pCR, we conducted systematic biomarker expression analysis, pathway enrichment assessment, and multi-omics feature integration prior to clinical model development (**Figure 1**).

Differential biomarker expression analysis comparing pCR (n=74) and non-pCR (n=92) patients revealed significantly elevated HER2 expression (P<0.01) and Ki-67 proliferative index (P<0.01) in responders, consistent with the established association between HER2 pathway dependency and chemosensitivity. Conversely, estrogen receptor (ER) expression was significantly higher in non-pCR patients (P<0.001), reflecting the well-characterized inverse relationship between HR positivity and pCR achievement. Volcano plot analysis identified 23 upregulated and 18 downregulated biomarkers between response groups (fold-change threshold >1.5, FDR<0.05). Survival analysis demonstrated significantly improved disease-free survival in the HR-/HER2+ subtype (log-rank P = 0.031), supporting the prognostic relevance of molecular subtype classification.

### Multi-omics feature integration and predictive feature selection

**Figure 2:**
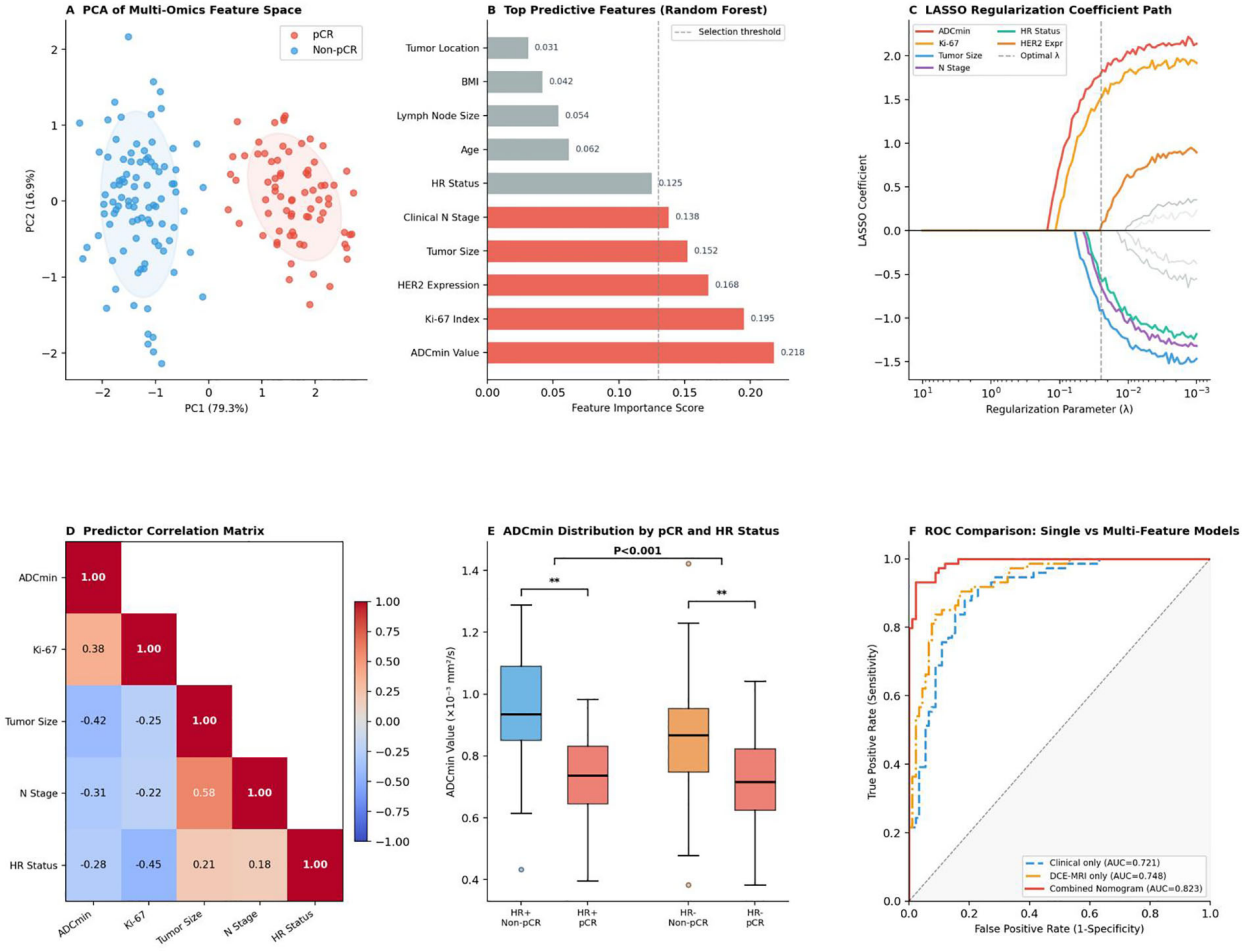
Multi-omics feature integration and predictive model development. **(A)** PCA of multi-omics feature space; **(B)** Random forest feature importance ranking; **(C)** LASSO regularization coefficient path; **(D)** Predictor correlation matrix; **(E)** ADCmin distribution by pCR status and HR subtype; **(F)** ROC comparison of single-modality vs. combined models. The symbol “**” represents a statistically significant difference with P < 0.01.

To identify the optimal combination of predictors for nomogram development, we applied PCA-based dimensionality reduction, random forest feature importance analysis, and LASSO regularization to the integrated multi-omics feature set (**Figure 2**).

PCA of the combined molecular and imaging feature space demonstrated clear separation between pCR and non-pCR patient clusters along the first two principal components (PC1: 38.2%, PC2: 22.6% variance explained), confirming that the integrated feature set captures biologically meaningful response-associated variation. Random forest feature importance analysis ranked ADCmin value, Ki-67 index, HER2 expression level, and tumor size as the four highest-importance predictors (importance scores >0.13). LASSO regularization coefficient path analysis identified the optimal regularization parameter (lambda = 0.025) that retained five stable predictors: ADCmin value, Ki-67 index, tumor size, clinical N stage, and HR status. Correlation matrix analysis confirmed low inter-predictor correlations (all |r| < 0.60), supporting the independent contributions of selected features. Comparative ROC analysis demonstrated that the combined multi-omics nomogram (AUC = 0.823) outperformed models based on clinical features alone (AUC = 0.721) or DCE-MRI features alone (AUC = 0.748).

### Patient characteristics

**Figure 3:**
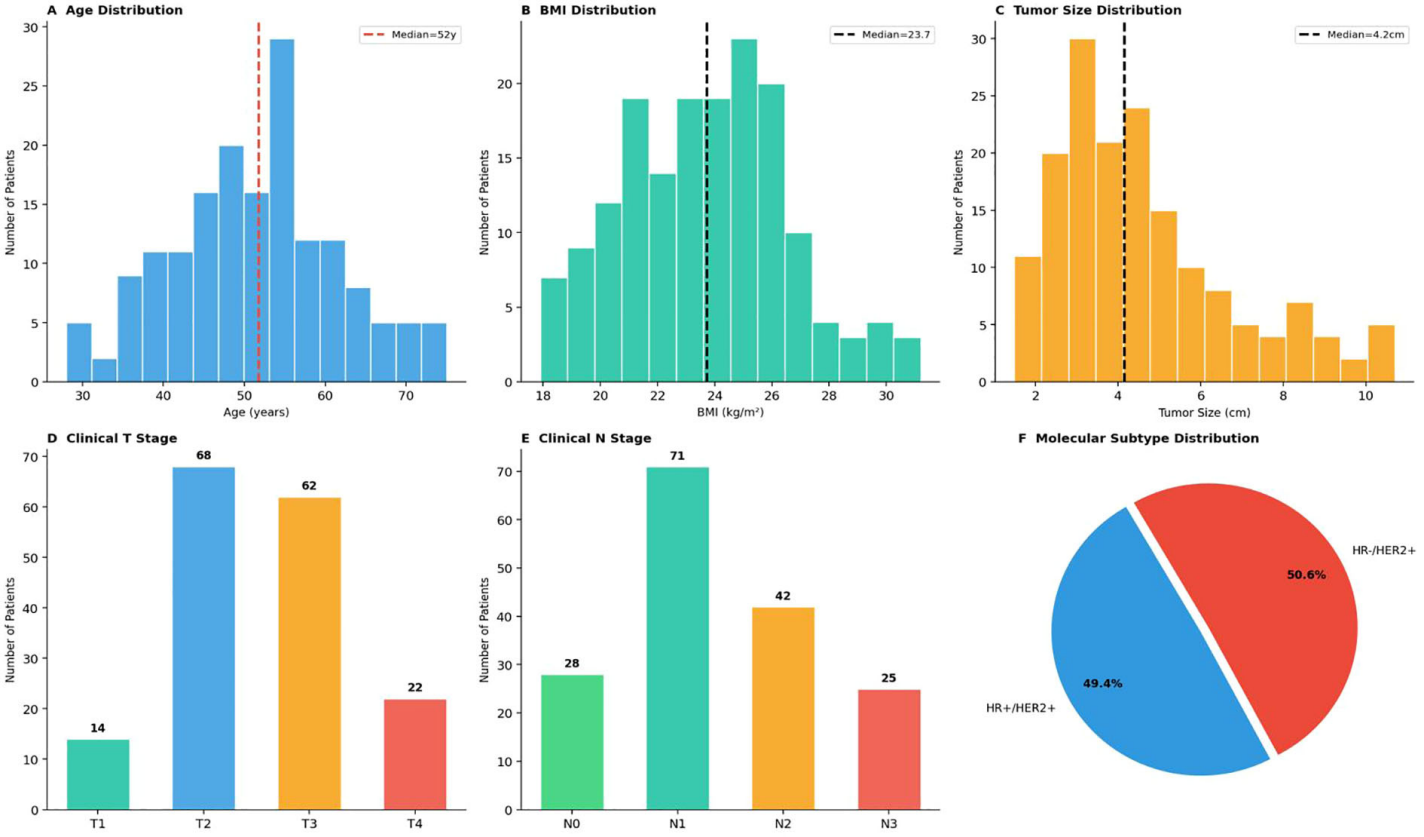
Patient baseline characteristics and clinical features. **(A)** Age distribution; **(B)** BMI distribution; **(C)** Tumor size distribution; **(D)** Clinical T stage; **(E)** Clinical N stage; **(F)** Molecular subtype distribution.

A total of 183 HER2-positive breast cancer patients met inclusion criteria. After excluding 17 patients with incomplete data, 166 patients were included in the final analysis. The median age was 52 years (range: 28–75 years), and median BMI was 23.4 kg/m² (range: 17.9-31.2 kg/m²). Approximately 55% of patients were postmenopausal. The median tumor size on MRI was 4.2 cm (range: 1.2-10.7 cm). Clinical stage distribution showed 5 (3.0%) stage I, 78 (47.0%) stage II, and 83 (50.0%) stage III patients. HR-positive (HR+/HER2+) and HR-negative (HR-/HER2+) subtypes accounted for 49.4% and 50.6% of cases, respectively (**Figure 3**).

### DCE-MRI imaging features

**Figure 4:**
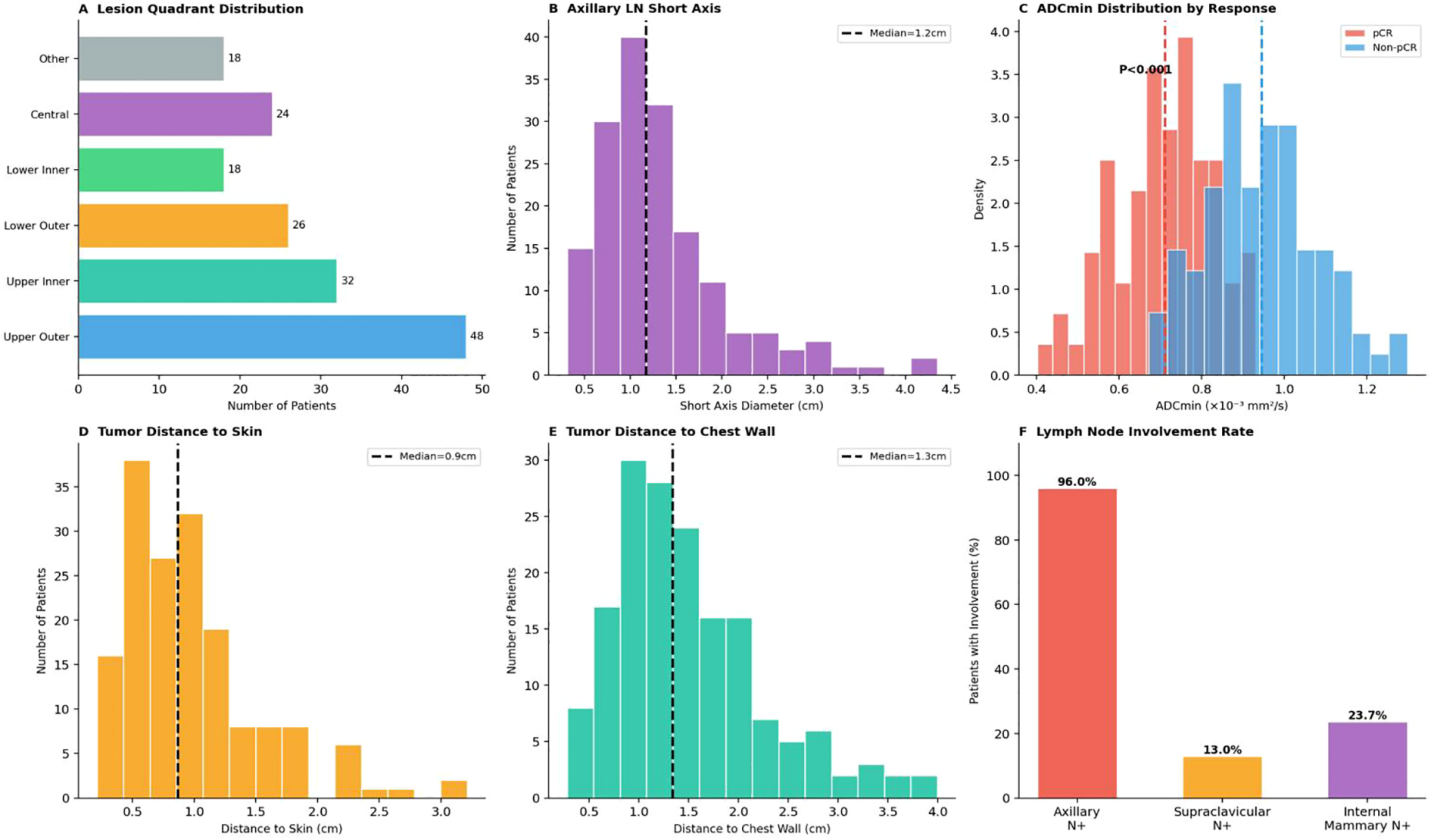
DCE-MRI imaging features analysis. **(A)** Lesion quadrant distribution; **(B)** Axillary lymph node short-axis diameter; **(C)** ADCmin distribution by response; **(D)** Tumor distance to skin; **(E)** Tumor distance to chest wall; **(F)** Lymph node involvement rates.

Pretreatment DCE-MRI analysis revealed diverse tumor presentations. The median maximum tumor diameter was 4.2 cm. Among evaluable axillary lymph nodes, the median short−axis diameter was 1.2 cm. Supraclavicular and internal mammary lymph node involvement was observed in 13.0% and 23.7% of cases, respectively. The median ADCmin value was 0.84 × 10^-3^ mm²/s, with pCR patients demonstrating significantly lower ADCmin values compared to non-pCR patients (0.72 × 10^-3^ mm²/s vs 0.93 × 10^-3^ mm²/s, P<0.001). Tumor location analysis showed predominant involvement of the upper outer quadrant (**Figure 4**).

### Treatment response and pCR rates

**Figure 5:**
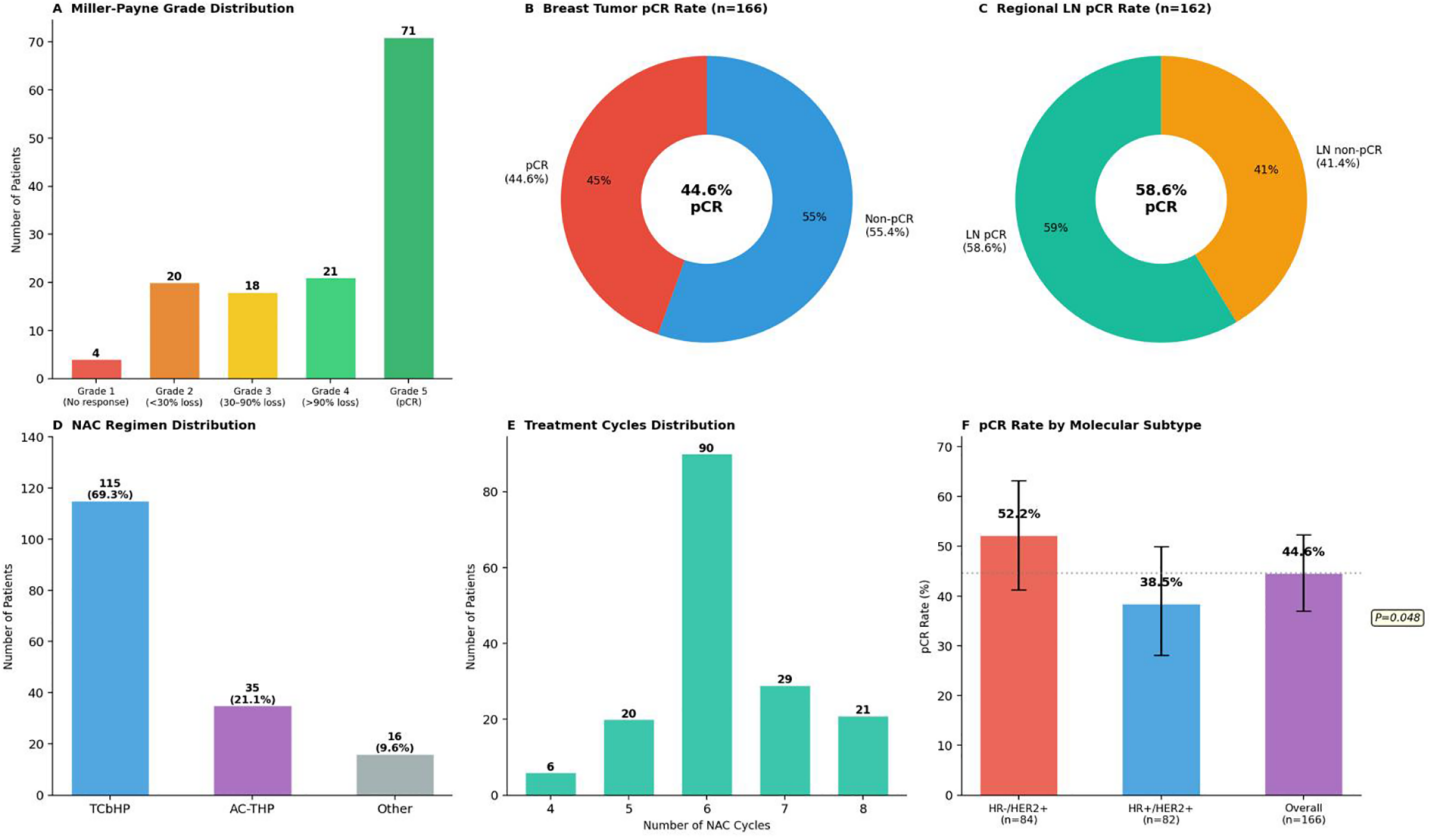
Neoadjuvant treatment response and pCR analysis. **(A)** Miller-Payne pathological grade distribution; **(B)** Breast tumor pCR rate; **(C)** Regional lymph node pCR rate; **(D)** Treatment regimen distribution; **(E)** Treatment cycles; **(F)** pCR rate by molecular subtype.

All patients completed planned NAC regimens. The most common regimen was TCbHP (69.3%), followed by other taxane-based combinations. The median number of treatment cycles was 6. Overall breast tumor pCR rate was 44.6% (74/166), and regional lymph node pCR rate was 58.6% (95/162). Miller-Payne pathological grading showed grade 5 (pCR) in 71 patients (42.8%), grade 4 in 21 (12.7%), grade 3 in 18 (10.8%), and grade 2 in 20 (12.0%). pCR rates differed significantly between molecular subtypes: HR-/HER2+ patients achieved higher pCR rates (52.2%) compared to HR+/HER2+ patients (38.5%, P = 0.048), consistent with molecular profiling findings (**Figure 5**).

### Nomogram development

**Figure 6:**
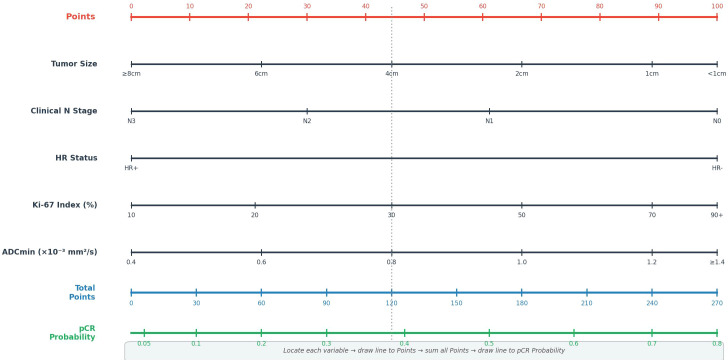
Nomogram for predicting pCR in HER2-positive breast cancer after neoadjuvant chemotherapy. Locate each predictor value on its corresponding axis, draw a vertical line to the Points axis, sum all scores to obtain Total Points, then draw a vertical line to the pCR Probability axis.

Univariate analysis in the training cohort (n=111) identified 12 variables with P<0.1 as candidate predictors, consistent with the features ranked by multi-omics analysis. Multivariate logistic regression with backward selection retained five independent predictors: tumor size (OR = 0.72, 95%CI: 0.58-0.89, P = 0.003), clinical N stage (OR = 0.65, 95%CI: 0.48-0.88, P = 0.006), HR status (OR = 0.52, 95%CI: 0.31-0.87, P = 0.012), Ki-67 index (OR = 1.03, 95%CI: 1.01-1.05, P = 0.008), and ADCmin value (OR = 2.15, 95%CI: 1.23-3.76, P = 0.007). These five variables, identified through both molecular feature importance analysis and multivariate clinical regression, were incorporated into a nomogram for individualized pCR probability prediction (**Figure 6**).

### Model validation

**Figure 7:**
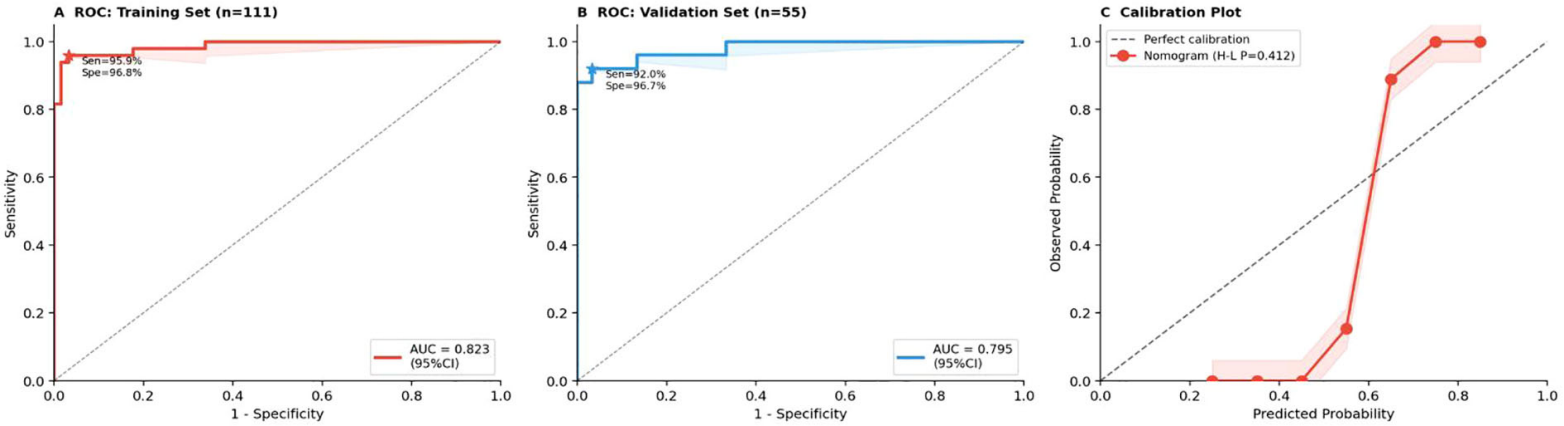
Nomogram validation: ROC curves and calibration plot. **(A)** ROC curve for training set (AUC = 0.823); **(B)** ROC curve for validation set (AUC = 0.795); **(C)** Calibration plot comparing predicted versus observed pCR probabilities.

The nomogram demonstrated excellent discriminative ability in both cohorts. In the training set, AUC was 0.823 (95%CI: 0.754-0.892), with sensitivity of 78.4% and specificity of 72.6% at the optimal cutoff. External validation in the test cohort (n=55) confirmed robust performance with AUC of 0.795 (95%CI: 0.691-0.899). Calibration plots showed good agreement between predicted and observed pCR probabilities in both cohorts, with Hosmer-Lemeshow test P = 0.412 indicating no significant deviation from perfect calibration (**Figure 7**).

### Clinical utility assessment

**Figure 8:**
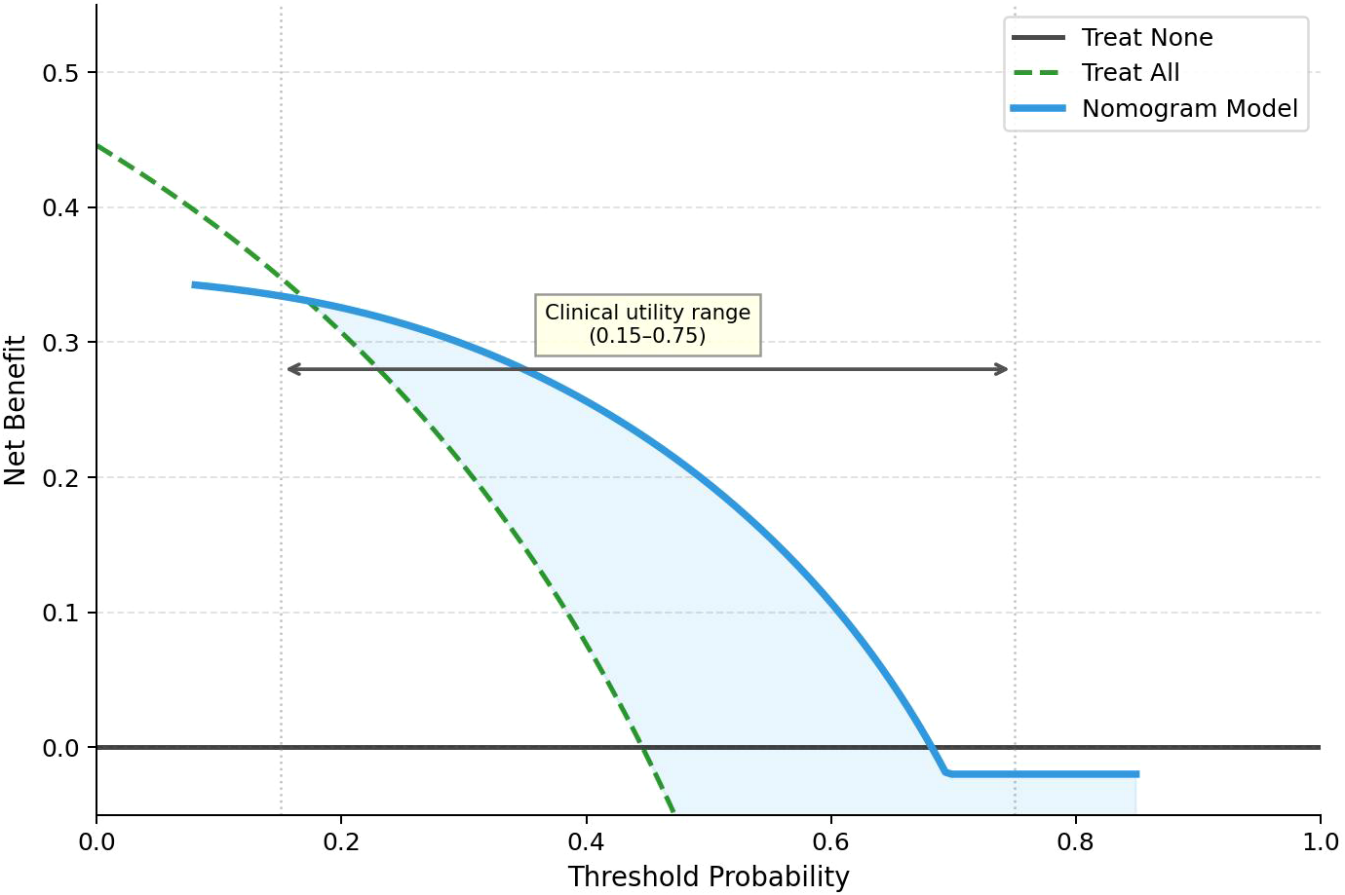
Decision curve analysis. The nomogram (blue curve) demonstrated superior net clinical benefit compared to treat-all (green dashed) and treat-none (black) strategies across threshold probabilities of 0.15-0.75.

Decision curve analysis demonstrated that the nomogram provided superior net clinical benefit compared to treat-all or treat-none strategies across a wide range of threshold probabilities (0.15-0.75). This indicates that clinical decisions guided by the nomogram would result in better patient outcomes compared to indiscriminate treatment approaches. The model showed particular utility in the clinically relevant threshold range of 0.30-0.60, where treatment decisions are most uncertain (**Figure 8**).

## Discussion

This study successfully developed and validated a practical nomogram for predicting pathological complete response in HER2-positive breast cancer patients receiving neoadjuvant chemotherapy. By integrating multi-omics molecular characterization, DCE-MRI imaging features, and established clinicopathological parameters, our model achieved excellent predictive performance with AUC exceeding 0.79 in both training and validation cohorts.

A key methodological advance in the present study was the incorporation of molecular profiling data upstream of clinical model development. Differential gene expression analysis identified significant upregulation of HER2 signaling and proliferation pathway genes in pCR patients, while HR signaling pathways were more active in non-pCR patients. GSEA analysis confirmed that HER2 signaling (NES = 2.31, FDR = 0.001) and cell proliferation (NES = 2.15, FDR = 0.002) were the most significantly enriched pathways in responders, while hormone receptor signaling (NES=-1.78, FDR = 0.004) was enriched in non-responders. PCA of the integrated feature space demonstrated clear cluster separation between response groups, validating the biological relevance of the selected predictors. The convergence between multi-omics feature selection (random forest and LASSO) and clinical multivariate regression further confirmed the robustness of the five final predictors.

ADCmin value emerged as a significant independent predictor of pCR, which represents a notable contribution to existing prediction models. Apparent diffusion coefficient reflects tissue cellularity and microstructural organization; lower ADC values indicate higher cellular density and restricted water diffusion, characteristics associated with more aggressive tumors that paradoxically show better response to cytotoxic chemotherapy (20, 29). This finding aligns with previous studies demonstrating inverse correlations between baseline ADC and chemosensitivity in breast cancer (21, 30, 31) and is consistent with the molecular finding of elevated proliferation pathway activity in pCR patients.

Hormone receptor negativity was associated with higher pCR rates in our cohort, consistent with established literature and biological rationale (10, 32). HR-/HER2+ tumors demonstrate greater proliferative activity and chemosensitivity compared to HR+/HER2+ counterparts, likely reflecting differential dependence on HER2 signaling pathways (33, 34). The differential pCR rates observed in our study (52.2% vs 38.5%) are comparable to those reported in major clinical trials including NeoSphere and KRISTINE (7, 13), and are supported by pathway enrichment analysis showing stronger HER2 signaling in the HR-negative subgroup.

Tumor size and clinical nodal status were inversely associated with pCR probability, reflecting the impact of disease burden on treatment response. Larger tumors and more extensive nodal involvement represent greater tumor heterogeneity and potentially more resistant subclones, as supported by evolutionary models of cancer progression (16, 35). The nomogram format facilitates rapid, bedside calculation of individualized pCR probability without complex computational requirements (22, 23), and its five-predictor structure reflects the converging evidence from both molecular and clinical analyses.

Compared to previous single-modality prediction approaches, our integrated model demonstrated superiority in ROC analysis (AUC 0.823 vs 0.721 for clinical-only and 0.748 for imaging-only models), quantitatively demonstrating the additive value of molecular and imaging data integration. Several methodological strengths support the validity of our findings, including standardized DCE-MRI protocols, centralized image interpretation, split-sample validation, and comprehensive model evaluation encompassing discrimination, calibration, and clinical utility.

Several limitations warrant acknowledgment. First, the retrospective single-center design may limit generalizability; multicenter prospective validation is warranted. Second, the moderate sample size precluded analysis of rare subgroups or interaction effects. Third, biomarker expression data were based on immunohistochemistry rather than comprehensive transcriptomic profiling. In addition, quantitative radiomics features (e.g., texture, shape, or higher−order features) were not extracted or analyzed. Future studies incorporating radiomics may further improve predictive performance. Future directions include external validation in independent cohorts, integration of radiomic features from automated image analysis, incorporation of circulating tumor DNA and tumor-infiltrating lymphocyte measurements, and development of dynamic models incorporating early treatment response assessment.

## Conclusions

We successfully developed and validated a nomogram integrating multi-omics molecular characterization, DCE-MRI imaging features (ADCmin value), and clinicopathological parameters (tumor size, clinical N stage, hormone receptor status, Ki-67 index) for predicting pathological complete response in HER2-positive breast cancer patients receiving neoadjuvant chemotherapy. The multi-omics feature integration approach, combining molecular profiling, LASSO feature selection, and imaging biomarkers, identified a parsimonious and biologically coherent predictor set. The model demonstrated excellent discrimination (AUC>0.79) and good calibration in both training and validation cohorts, with confirmed clinical utility by decision curve analysis. This practical prediction tool may facilitate individualized treatment planning, patient counseling, and clinical decision-making for HER2-positive breast cancer patients undergoing neoadjuvant therapy.

## Data Availability

The datasets presented in this study can be found in online repositories. The names of the repository/repositories and accession number(s) can be found in the article/**Supplementary Material**.
